# Combining liquid biopsy and functional imaging analysis in metastatic castration‐resistant prostate cancer helps predict treatment outcome

**DOI:** 10.1002/1878-0261.13120

**Published:** 2021-11-09

**Authors:** Vincenza Conteduca, Emanuela Scarpi, Paola Caroli, Cristian Lolli, Giorgia Gurioli, Nicole Brighi, Giulia Poti, Alberto Farolfi, Amelia Altavilla, Giuseppe Schepisi, Federica Matteucci, Giovanni Paganelli, Ugo De Giorgi

**Affiliations:** ^1^ Department of Medical Oncology IRCCS Istituto Romagnolo per lo Studio dei Tumori (IRST) “Dino Amadori” Meldola Italy; ^2^ Unit of Medical Oncology and Biomolecular Therapy Department of Medical and Surgical Sciences University of Foggia, Policlinico Riuniti Italy; ^3^ Unit of Biostatistics and Clinical Trials IRCCS Istituto Scientifico Romagnolo per lo Studio dei Tumori (IRST) “Dino Amadori” Meldola Italy; ^4^ Nuclear Medicine Operative Unit IRCCS Istituto Scientifico Romagnolo per lo Studio dei Tumori (IRST) “Dino Amadori” Meldola Italy; ^5^ Biosciences Laboratory IRCCS Istituto Scientifico Romagnolo per lo Studio dei Tumori (IRST) “Dino Amadori” Meldola Italy; ^6^ Istituto Dermopatico dell'Immacolata IDI‐IRCCS Rome Italy

**Keywords:** choline PET/TC, metabolic activity, metastatic castration‐resistant prostate cancer, plasma tumour DNA, prognosis

## Abstract

Plasma tumour DNA (ptDNA) is a potential early noninvasive biomarker of treatment outcome in metastatic castration‐resistant prostate cancer (mCRPC). Herein, we investigated whether pretreatment ptDNA levels reflect metabolic tumour burden in mCRPC and better predict treatment outcome in combination with functional imaging. Targeted next‐generation sequencing was performed to estimate the ptDNA fraction from 102 mCRPC patients receiving abiraterone or enzalutamide. The maximum standardized uptake value (SUVmax), total lesion activity (TLA) and metabolic tumour volume (MTV) were evaluated on ^18^F‐fluorocholine positron emission tomography/computed tomography. We assessed a Weibull multiple regression model to determine the combined impact of clinical, molecular and imaging characteristics on overall survival (OS) and progression‐free survival (PFS), and to obtain prognostic scores. A significant association was seen between ptDNA and SUVmax, MTV and TLA. For survival analysis, patients were randomly allocated into a training (*n* = 68) and a validation (*n* = 34) set. In the training set, multivariable analyses showed that ptDNA, MTV and serum lactate dehydrogenase together with visceral metastasis were independent predictors of both OS and PFS. Prognostic scores were generated, with the identification of three groups of patients with significantly different median OS (29.2, 15.9 and 8.7 months) and PFS (13.3, 7.7 and 3.2 months) probabilities. The differences in median survival between risk groups were confirmed in the validation cohort for both OS and PFS. In our study, we showed that integrating plasma DNA analysis with functional imaging may improve prognostic risk stratification and treatment selection in mCRPC.

## Graphical abstract

Plasma tumour DNA (ptDNA) has been recently demonstrated as a potential early noninvasive biomarker in patients affected with metastatic castration‐resistant prostate cancer receiving androgen receptor signalling inhibitors. In our study, we initially showed that ptDNA reflected tumour metabolic activity. Additionally, integrating ptDNA analysis with functional imaging and clinical features showed potential for an improved outcome prediction as well as for a better treatment selection in these patients.

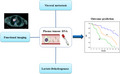

**Abbreviations**
ALP: alkaline phosphatase
AR: androgen receptor
ARSI: androgen receptor‐signalling inhibitors
CGA: chromogranin A
CI: confidence interval
CT: computed tomography
CTC: circulating tumour cell
ECOG: Eastern Cooperative Oncology Group
HR: hazard ratio
LDH: lactate dehydrogenase
mCRPC: metastatic castration‐resistant prostate cancer
MTV: metabolic tumour volume
OS: overall survival
PCWG3: Prostate Cancer Working Group 3
PET/CT: positron emission tomography/computed tomography
PFS: progression‐free survival
PSA: prostate‐specific antigen
ptDNA: plasma tumour DNA
SUVmax: maximum standardized uptake value
TLA: total lesion activity

## 1 Introduction

Prostate cancer is one of the most frequent malignancies in male adults [1]. Like many cancers, prostate cancer is heterogeneous and reveals a branched evolutionary pattern going from locally advanced to progression of metastatic tumour [2]. Furthermore, clonal evolution continues to happen as a result of systemic therapy, with selection for diverse resistance mechanisms, including reactivation of the androgen–androgen receptor (AR) axis via *AR* gene aberrations such as amplification, mutations and variants upon challenging of AR signalling inhibitors (ARSI) [3, 4, 5, 6].

In men with prostate cancer receiving a systemic treatment, tumour burden and responses to therapy are commonly assessed through clinical evaluation, prostate‐specific antigen (PSA) levels and radiologic imaging with computed tomography (CT), bone scan, magnetic resonance imaging and positron emission tomography (PET) [7]. Significant efforts have been directed towards developing and enhancing imaging methods for the characterization of prostate tumours. CT/PET offers the opportunity to assess both tumour volume and metabolic disease activity. Despite reliability of current imaging techniques, complementary strategies are warranted to better guide treatment choices.

Liquid biopsy has established itself as method able to overcome the inherent challenges of following treatment resistance using tissue biopsies. Contrary to a tissue biopsy, a liquid biopsy is minimally invasive, capable of real‐time molecular characterization of the disease through the identification of plasma tumour DNA (ptDNA) and able to capture a composite picture of intra‐ and intertumour heterogeneity of the potentially clinically relevant disease [8, 9].

Our group has previously shown that positron emission tomography/computed tomography (PET/CT) with ^18^F‐fluorocholine (FCH) can be used for early response assessment in metastatic castration‐resistant prostate cancer (mCRPC) patients treated with abiraterone and enzalutamide [10, 11] and that combining PET/CT with *AR* copy number gain detected in ptDNA associate with outcome in mCRPC [12]. However, the association between ptDNA fraction, as an early noninvasive biomarker of treatment response [3, 13, 14, 15], and tumour burden remains largely unexplored. In this study, we sought to establish the relationship between ptDNA fraction and metabolic tumour burden and if ptDNA in combination with functional imaging more accurately predicts treatment outcome in mCRPC.

## 2 Materials and methods

### 2.1 Study design

In this work, we evaluated mCRPC patients with histologically confirmed diagnosis of prostate adenocarcinoma without evidence of neuroendocrine differentiation and small cell histology. Selection criteria included also patients with serum testosterone < 50 ng·dL^−1^ with androgen deprivation therapy and Eastern Cooperative Oncology Group (ECOG) performance status ≤ 2. Patients received abiraterone 1000 mg with twice‐daily prednisone 5 mg or enzalutamide 160 mg once daily until evidence of progressive disease or unacceptable toxicity.

Peripheral blood samples for plasma DNA analysis, laboratory parameters such as complete blood count, PSA, serum lactate dehydrogenase (LDH), alkaline phosphatase (ALP) and chromogranin A (CGA), were assessed within 1 week of therapy initiation. FCH‐PET/CT imaging was performed within 1 week before starting ARSI therapy. PSA response and toxicity were monthly assessed. Radiographic evaluation was performed with the use of CT scan before treatment and every 3 months thereafter as for institutional clinical practice. Response was retrospectively assessed according to Prostate Cancer Working Group 3 (PCWG3) guidelines [7], and soft tissue disease was evaluated on the basis of Response Evaluation Criteria in Solid Tumours (RECIST) version 1.1

The study was conducted in accordance with the Declaration of Helsinki and the Good Clinical Practice guidelines of the International Conference of Harmonization. The Institutional Review Board approved biomarker study (REC 2192/2013), and patients signed informed consent.

### 2.2 PET/CT imaging protocol

FCH‐PET/CT scans were carried out on an integrated PET/CT system (Discovery LS camera; General Electric Medical Systems, Waukesha, WI, USA) in 2D acquisition mode for 3 min per bed position. The PET/CT scan takes 45 min after intravenous injection of ^18^F‐methylcholine (3.7 MBq·kg^−1^ of body weight, AAA‐Advanced Accelerator Applications, Meldola, Italy). The field of view included the skull to mid‐femurs. Low dose CT (120 kV, 80 mA) without contrast agents was made for attenuation correction and as an anatomical map. The emission data were adjusted for scatter, random coincidence events, and system dead time. Two nuclear medicine physicians with good experience did the reading and interpretation of FCH‐PET/CT scans. Criteria to identify FCH‐PET/CT positivity consisted of the presence of focal areas of raised tracer uptake with or without any underlying lesion found by performing CT. Semiquantitative criteria based on the maximum standardized uptake value (SUVmax) and the target‐to‐background ratio were utilized to aid the visual analysis [16]. The metabolic tumour volume (MTV) parameter was obtained by adding each three‐dimensional volume of interest, and for each lesion volume and SUV mean was multiplied and then summed to have the total lesion activity (TLA). MTV is only a volumetric entity, while TLA takes also into account the metabolic activity of the lesion, so providing an evaluation of the tumour activity. FCH‐PET/CT scans were read sequentially thanks to a Xeleris III Workstation (General Electric Medical Systems). PET, CT and PET/CT fused images supply to provide the scans in axial, sagittal and coronal sections.

### 2.3 Plasma tumour DNA analysis

Cell‐free DNA was extracted from 1 to 2 mL of plasma with the QIAamp Circulating Nucleic Acid Kit (Qiagen, Santa Clarita, CA, USA) and quantified by spectrophotometric evaluation (NanoDrop^®^ ND‐1000; Celbio, Milan, Italy) or Quant‐iT High Sensitivity PicoGreen Double‐Stranded DNA Assay Kit (Invitrogen, Carlsbad, CA, USA). In plasma and patient‐matched germline DNA, targeted next‐generation sequencing (NGS) was assessed by the PGM Ion Torrent using a 316 or 318 Chip aiming to reach 1000× coverage per target. We estimated the plasma tumour fraction and *AR* gene copy number for each plasma sample from the study patients by using the approach previously described [3, 4].

### 2.4 Statistical analysis

Progression‐free survival (PFS) was considered as the time between the first day of ARSI therapy and the date of progression disease or death (whichever came first). Men who had not progressed at database closure were censored at the last tumour assessment or treatment discontinuation for severe adverse events. Overall survival (OS) was considered as the time between the first day of ARSI treatment and the date of death from any cause or the date of the last follow‐up visit.

Categorical variables were summarized using frequency whereas continuous variables were described using median value and interquartile range. The association between categorical variables was determined using the chi‐squared or Fisher's exact test, as appropriate. Spearman correlation was used to assess the association between continuous variables.

Survival curves were estimated by the Kaplan–Meier method, and comparisons were made using the logrank test. Univariable and multivariable Cox regression models were utilized to explore potential factors able to predict PFS and OS and to estimate hazard ratios (HRs) and their 95% confidence interval (CI). All *P*‐values were two‐sided, and a *P* < 0.05 was defined as statistically significant. Statistical analyses were done with sas 9.4 software (SAS Institute, Cary, NC, USA).

We used a Weibull multiple regression model to assess the matched impact of molecular, laboratory and imaging characteristics on outcome. From a full model including these factors, we achieved a final parsimonious model by using a backward selection procedure. The prognostic score was built on the final model consisting of four factors for OS and three for PFS. Partial scores were procured by splitting the value of each regression coefficient by the smallest regression coefficient. The total score for each patient resulted from a sum of appropriate partial scores, and three patient groups with different median survival probabilities were recognized. For OS, if the total score was 1 or below, between 1.1 and 2.5, and > 2.5, patients were classified as group I, group II and group III, respectively. For PFS, if the total score was 1 or below, between 1.0 and 2.1, and > 2.1, patients were classified as group I, group II and group III.

## 3 Results

### 3.1 Patient cohort characteristics

Between October 2011 and June 2016, 102 plasma samples were collected from CRPC patients at start of treatment with ARSI. Sixty‐six patients were treated with abiraterone, 36 with enzalutamide, 27 (26.5%) were chemotherapy‐naïve, 75 (73.5%) were previously chemotherapy‐treated, and 17 (16.7%) received prior therapy with ARSI. Interactions between ARSI treatment and ptDNA analysed in the Cox models were *P* = 0.073 for OS and *P* = 0.213 for PFS.

Patients treated with ARSI were randomly splitted into one training set (*n* = 68) and one validation set (*n* = 34). The larger two‐third group of men was utilized as a training set to build the prognostic score, and the remaining one‐third was considered as a validation cohort. Except for lower ALP concentration in the training vs. validation cohort (*P* = 0.0002), no significant differences in baseline characteristics were seen between training and validation groups (Table 1).

**Table 1:** Patient characteristics. *n*, number; NLR, neutrophil–lymphocyte ratio; PS, performance status; SUV, standardized uptake value.

	Total (*n* = 102)	Training (*n* = 68)	Validation (*n* = 34)	*P*
	*n* (%)	*n* (%)	*n* (%)	
Age, years				
≤ 74^a^	54 (52.9)	40 (58.8)	14 (41.2)	
> 74	48 (47.1)	28 (41.2)	20 (58.8)	0.092
Prostatectomy				
No	60 (58.8)	40 (58.8)	20 (58.8)	
Yes	42 (41.2)	28 (41.2)	14 (41.2)	1.000
Radical radiotherapy				
No	58 (56.9)	42 (61.8)	16 (47.1)	
Yes	44 (43.1)	26 (38.2)	18 (52.9)	0.157
Gleason score				
6–7	41 (45.6)	28 (45.9)	13 (44.8)	
8–10	49 (54.4)	33 (54.1)	16 (55.2)	0.924
Site of metastasis				
Bone	91 (89.2)	63 (92.7)	28 (82.3)	0.173
Lymph nodes	53 (52.0)	37 (54.4)	16 (47.1)	0.483
Liver	5 (4.9)	3 (4.4)	2 (5.9)	0.746
Lung	9 (8.8)	8 (11.8)	1 (2.9)	0.265
ECOG PS				
0–1	99 (97.1)	66 (97.1)	33 (97.1)	
≥ 2	3 (2.9)	2 (2.9)	1 (2.9)	1.000
Presence of pain				
No	94 (92.2)	62 (91.2)	32 (94.1)	
Yes	8 (7.8)	6 (8.8)	2 (5.9)	0.602
Type of treatment				
Abiraterone	66 (64.7)	46 (67.7)	20 (58.8)	
Enzalutamide	36 (35.3)	22 (32.3)	14 (41.2)	0.379
Chemotherapy‐naive				
No	75 (73.5)	51 (75.0)	24 (70.6)	
Yes	27 (26.5)	17 (25.0)	10 (29.4)	0.634
Prior therapeutic lines				
1–2	65 (63.7)	45 (66.2)	20 (58.8)	
> 2	37 (36.3)	23 (33.8)	14 (41.2)	0.466
Serum LDH, U·L^−1^				
< 225^b^	78 (76.5)	50 (73.5)	28 (82.3)	
≥ 225	24 (23.5)	18 (26.5)	6 (17.7)	0.322
ALP, U·L^−1^				
< 129^b^	67 (65.7)	53 (77.9)	14 (41.2)	
≥ 129	35 (34.3)	15 (22.1)	20 (58.8)	0.0002
NLR				
< 3^b^	54 (52.9)	35 (51.5)	19 (55.9)	
≥ 3	48 (47.1)	33 (48.5)	15 (44.1)	0.674
Serum CGA, ng·mL^−1^				
< 120^b^	48 (47.1)	29 (42.7)	19 (55.9)	
≥ 120	54 (52.9)	39 (57.3)	15 (44.1)	0.207
Haemoglobin, g·dL^−1^				
> 12.5^b^	40 (39.2)	25 (36.8)	15 (44.1)	
≤ 12.5	62 (60.8)	43 (63.2)	19 (55.9)	0.473
Serum albumin, g·dL^−1^				
> 4^b^	50 (52.6)	32 (50.8)	18 (56.2)	
≤ 4	45 (47.4)	31 (49.2)	14 (43.8)	0.615
Unknown/missing	7	5	2	
Serum PSA, ng·dL^−1^				
< 23.24^a^	50 (49.5)	32 (47.8)	18 (52.9)	
≥ 23.24	51 (50.5)	35 (52.2)	16 (47.1)	0.623
Unknown/missing	1	1	0	
Number of lesions on FCH‐CT/PET				
< 12^a^	51 (50.0)	33 (48.5)	18 (52.9)	
≥ 12	51 (50.0)	35 (51.5)	16 (47.1)	0.674
SUVmax				
< 83.60^a^	50 (49.5)	33 (49.2)	17 (50.0)	
≥ 83.60	51 (50.5)	34 (50.8)	17 (50.0)	0.943
Unknown/missing	1	1	0	
MTV				
< 102.79^a^	51 (50.0)	35 (51.5)	16 (47.1)	
≥ 102.79	51 (50.0)	33 (48.5)	18 (52.9)	0.674
TLA				
< 391343^a^	51 (50.0)	34 (50.0)	17 (50.0)	
≥ 391343	51 (50.0)	34 (50.0)	17 (50.0)	1.000
ptDNA				
≤ 0.188^a^	51 (50.0)	33 (48.5)	18 (52.9)	
> 0.188	51 (50.0)	35 (51.5)	16 (47.1)	0.674
*AR* copy number				
Normal	75 (73.5)	51 (75.0)	24 (70.6)	
Gain	27 (26.5)	17 (25.0)	10 (29.4)	0.634

a Median value.

b Upper normal value.

### 3.2 Associations of ptDNA with clinical variables and functional imaging

**Fig. 1:**
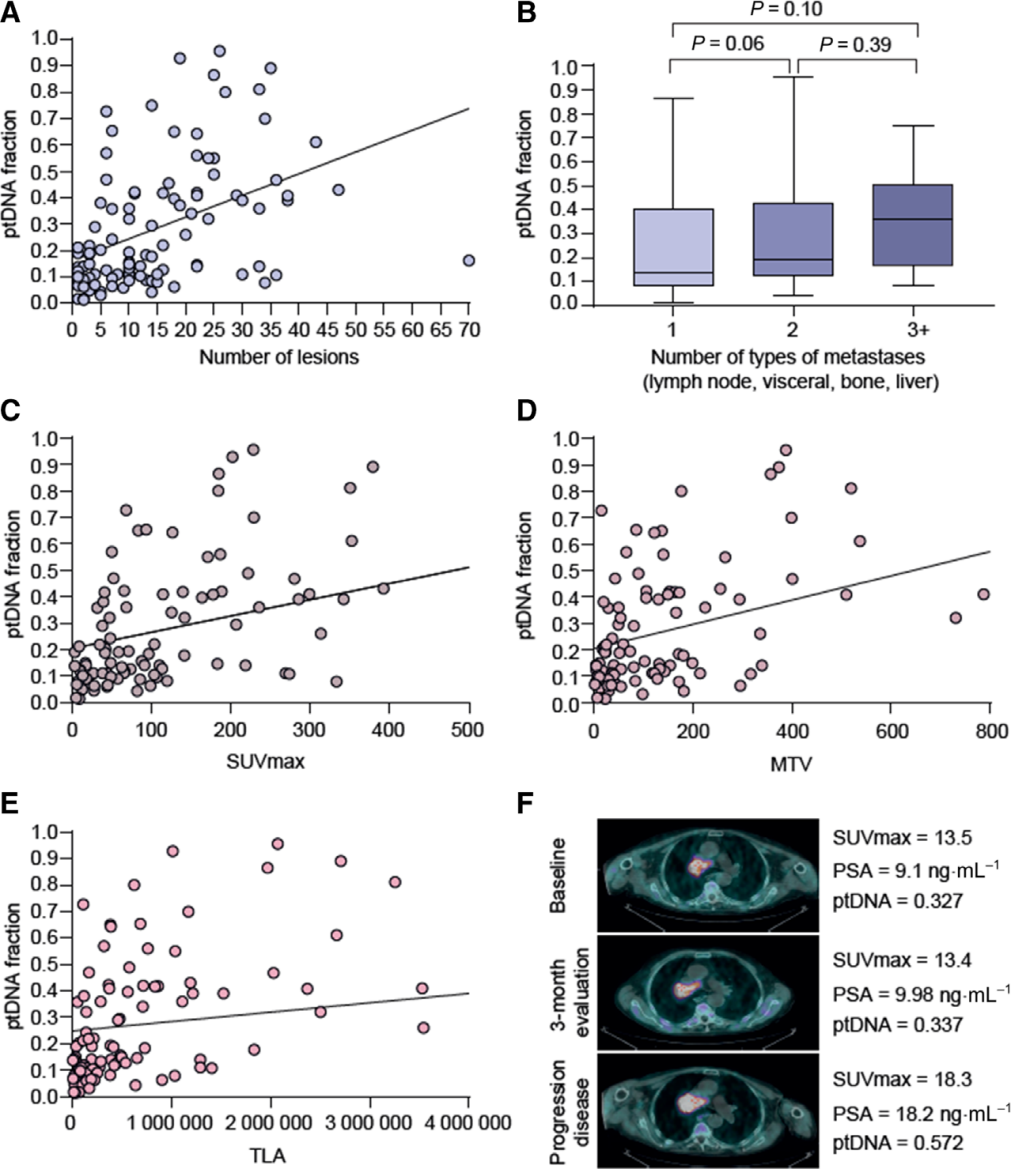
Association of ptDNA fraction with metastatic sites and metabolic activity. (A) Correlation of the number of metastatic sites and ptDNA. The outcome was the relationship between quantitative variables that was examined using the linear correlation coefficient (Pearson product moment correlation coefficient), *r* = 0.46, *P* < 0.0001. (B) Association of median ptDNA fraction and the number of types of metastases (66 patients had only one metastatic site, 54 had two metastatic sites, and five had more than two metastatic sites of disease). Box␣plot error bars show the range of the data set. All reasonably ‘extreme’ data are contained between the two ends of the error bars. Error bars are typically extended to be 1.5 times the interquartile range beyond the first and third quartiles if outlier values are present. We used Wilcoxon–Mann–Whitney test for comparison of ptDNA fraction considered as continuous data and number of types of metastases (two independent groups, *P* = 0.06 and *P* = 0.39) or Kruskal–Wallis test (three independent groups, *P* = 0.10). SUVmax (C), MTV (D) and TLA (E) associated with ptDNA fraction. The outcome was the relationship between quantitative variables that was examined using the Pearson linear correlation coefficient: (C) *r* = 0.48, *P* < 0.0001; (D) *r* = 0.47, *P* < 0.0001; (E) *r* = 0.37, *P* < 0.0001. (F) Representative case of association of metabolic activity and ptDNA fraction. MTV, metabolic tumour activity; TLA, tumour lesion activity.

Median fraction of ptDNA before starting treatment was 0.188 (0.014–0.96). We evaluated the distribution of tumour content fraction in plasma among patients with different number and types of metastasis. A significant correlation was reported between ptDNA and number of lesions (rs = 0.53, *P* < 0.0001) (Fig. 1A). However, ptDNA did not significantly associate with the number of different types of sites of metastasis (Fig. 1B) nor the specific type of metastasis a patient harboured although there was a trend for patients with liver metastasis (*P* = 0.058) or bone metastasis (*P* = 0.104) to have a higher ptDNA fraction (Fig. S1).

In addition, we correlated ptDNA and clinical variables at plasma collection and observed that ptDNA levels were significantly associated with LDH, ALP and PSA (Fig. S2). We also correlated *AR* copy number gain, detected in cell‐free DNA from 27.3% of overall patients, with ptDNA level showing a significant correlation (rs = 0.45, *P* < 0.0001) (Fig. S2).

Furthermore, we investigated the association between choline uptake measured as median SUVmax, MTV, TLA and ptDNA levels. As expected, FCH‐PET/CT parameters (SUVmax, MTV and TLA) were correlated with each other (Fig. S3). As it is known that FCH‐PET/CT is not an appropriate method for the evaluation of the liver status because of the normal tracer distribution [17], we excluded five patients harbouring hepatic metastases from all analyses of the current study (Table S1).

We reported a meaningful correlation between ptDNA fraction and choline uptake measured by SUVmax (rs = 0.544, *P* < 0.0001), MTV (rs = 0.488 *P* < 0.0001) and TLA (rs = 0.538 *P* < 0.0001) (Fig. 1C–E). A representative case, of a mCRPC postdocetaxel patient treated with abiraterone, illustrates this direct relationship between ptDNA and choline uptake on FCH‐PET/CT (Fig. 1F).

### 3.3 Ability of combining ptDNA analysis and functional imaging to predict clinical outcome

Median follow‐up was 49 months (range 1–50). The median OS and PFS for the overall population were 15.3 months (95% CI 11.4–17.5) and 5.7 months (95% CI 5.0–6.7), respectively. Recognizing the need for a prognostic tool that mirrors outcomes from currently available treatments, we examined variables associated with OS and PFS. Thus, we developed index models based on clinical, molecular and imaging factors.

In the training cohort, we performed univariate (Tables S2 and S3) and multivariable (Tables 2 and 3) Cox proportional hazards regression analyses of OS and PFS, respectively, to assess the associations between functional imaging, clinical features and molecular biomarkers and to generate prognostic scores.

**Table 2:** Multivariable analysis of OS after backward stepwise procedure in the training cohort. Total score ranges from 1 to 5.2.

	Factor estimate (standard error)	Standard error	*P*	HR (95% CI)	Partial score
MTV	0.599	0.268	0.026	1.82 (1.08–3.08)	1.00
ptDNA	0.848	0.289	0.003	2.34 (1.32–4.12)	1.40
Visceral metastasis	1.033	0.383	0.007	2.81 (1.33–5.95)	1.70
Serum LDH, U·L^−1^	1.239	0.331	0.0002	3.45 (1.81–6.60)	2.10

Risk groups	No. pts (%)	Total score
I	22 (33.9)	< 1.4
II	24 (36.9)	1.4–2.8
III	19 (29.2)	≥ 2.8

**Table 3:** Multivariable analysis of PFS after backward stepwise procedure in the training cohort. Total score ranges from 0 to 5.85.

	Factor estimate (standard error)	Standard error	*P*	HR (95% CI)	Partial score
MTV	0.586	0.271	0.031	1.80 (1.06–3.06)	1.00
ptDNA	0.645	0.266	0.015	1.91 (1.13–3.21)	1.10
Visceral metastasis	0.997	0.424	0.019	2.71 (1.18–6.22)	1.70
Serum LDH, U·L^−1^	1.204	0.323	0.0002	3.33 (1.77–6.27)	2.05

Risk groups	No. pts (%)	Total score
I	15 (23.1)	< 1.0
II	34 (52.3)	1.0–2.1
III	16 (24.6)	> 2.1

Multivariable analysis of OS after a backward stepwise procedure demonstrated that the presence of MTV, ptDNA, visceral metastasis and pretreatment serum LDH were significantly associated with OS [HR 1.82, 95% CI 1.08–3.08, *P* = 0.026; HR 2.34, 95% CI 1.32–4.12, *P* = 0.0003; HR 2.81, 95% CI 1.33–5.95, *P* = 0.007; and HR 3.45, 95% CI 1.81–6.60, *P* = 0.0002, respectively]. These variables were used to establish a prognostic index model of OS in mCRPC.

Table 2 presents the partial score value for category, originating from the subdivision of each regression coefficient by the smallest one as described in Statistical analysis. The total score for each patient was resulted from each patient's appropriate partial scores, and individuals with different median survival probabilities were classified into three groups. If the total score was equal to 1.4 or lower, the patient was allocated in group I, group II was characterized by a score between 1.4 and 2.8, and group III required a score > 2.8. The choice of time and number of groups with a different survival was more than 70% (group II, 15 months: OS was 30–70%; group III, 15 months: OS was < 30%). In the training set, 22 patients (33.9%) were assigned to risk group I, 24 (36.9%) to risk group II and 19 (29.2%) to risk group III.

**Fig. 2:**
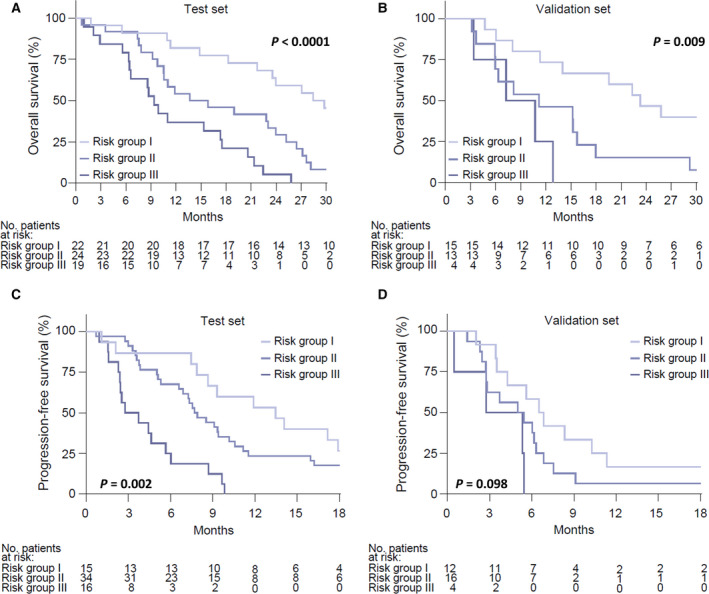
Risk group survival probabilities. Kaplan–Meier curves for OS by OS risk groups in the training set (A) and validation set (B) and PFS by PFS risk groups in the training set (C) and validation set (D). Survival curves were compared using Logrank test.panel. Pearson linear correlation coefficient: (A) *P* < 0.0001; (B) *P* = 0.009; (C) 0.002; (D) *P* = 0.098.

The survival experience of the three patient categories was established by the score. Survival probabilities were assessed by the exponential model and by the Kaplan–Meier method. In the training set, we observed a different median OS among the three risk groups (risk group I, 29.2 months [95% CI, 18.3–37.0 months]; risk group II, 14.8 months [95% CI, 9.8–24.0 months]; and risk group III, 9.4 months [95% CI, 6.3–17.4 months]; *P* < 0.0001) (Fig. 2A). Similarly, in the validation set, we showed risk group I, 23.4 months (95% CI, 8.1–38.5 months); risk group II, 11.2 months (95% CI, 6.0–15.8 months); and risk group III, 9.0 months (95% CI, 3.4–12.9 months); *P* = 0.009 (Fig. 2B).

In addition, MTV, ptDNA fraction, lung metastasis and serum LDH harboured independent value for PFS [HR 1.80, 95% CI 1.06–3.06, *P* = 0.031; HR 1.91, 95% CI 1.13–3.21, *P* = 0.015, HR 2.71, 95% CI 1.18–6.22, *P* = 0.019; and HR 3.33, 95% CI 1.77–6.27, *P* = 0.0002, respectively] (Table 3). Using Cox coefficient analysis of these parameters, we also established three prognostic groups with differing PFS (median, 13.3 vs. 7.7 vs. 3.2 months; *P* < 0.0001) (Fig. 2C), which was confirmed in the validation set (median, 6.6 vs. 5.1 vs. 4.0 months; *P* = 0.037) (Fig. 2D).

## 4 Discussion

Over the last decades, several prognostic scores have been developed by integrating different clinical variables and associating them with prognosis in mCRPC patients receiving standard chemotherapy and ARSI [18, 19, 20, 21, 22, 23, 24, 25]. The majority of these nomograms were characterized by the absence of predictive discrimination to support treatment selection, often limiting their clinical usefulness. Recent biomarker studies aimed to increase the prognostic and predictive value with the inclusion of AR‐V7 expression in circulating tumour cell (CTC), high circulating tumour content and many genetic aberrations involving *AR*, *TP53*, the PI3K/AKT pathway and homologous recombination repair [4, 5, 6, 26, 27]. Their integration with typical clinical variables in future models could increase accuracy and improve outcomes prediction, as highlighted herein through the combination of ptDNA analysis and functional imaging.

In our study, particularly striking was the association of ptDNA fraction with metabolic tumour activity and the number of lesions rather than the specific type of lesion, as shown in previous NGS studies on plasma samples from mCRPC and other clinical variants of prostate cancer [28, 29], suggesting that ptDNA fraction may provide interesting aspects of tumour biology and disease volume that are not captured by common clinical factors. Interestingly, despite these associations, both ptDNA and metabolic tumour activity were independent predictors of clinical outcomes in multivariate regression models. Consequently, metabolic tumour burden in combination with ptDNA analysis has the potential to ameliorate the accuracy of tumour response prediction and prognostication in mCRPC patients. In support of our findings, a recent prospective trial [30] also showed the utility of integrating functional imaging using ^18^F‐NaF PET/CT scan and the analysis of CTCs in mCRPC patients treated with enzalutamide. The authors showed that individual metastatic sites may show differential AR and AR‐V7 expression as well as neuroendocrine markers, and can be responsible of a heterogeneous response to enzalutamide as assessed by quantitative total bone imaging. Furthermore, De Laere *et al*. [31] developed a three‐stratum risk stratification system, using both clinical features and *TP53*‐alteration status in liquid biopsy to stratify patients in good and poor prognostic subgroups treated with ARSI. The current work builds on our previous discoveries describing *AR* copy number detected in plasma, by using digital droplet PCR, as a possible biomarker to generate a novel survival model [32]. In the study, circulating *AR* copy number, FCH‐PET parameters and other clinical factors were significant predictors of OS in mCRPC treated with ARSI and similar to this study could classify men into three risk groups thanks to the independent prognostic value of these biomarkers. In the present study, we included 65 patients previously investigated [32] with updated survival data and only those with plasma DNA suitable for tumour content detection. Altogether, these findings show that liquid biopsy analysis represents a noninvasive complementary tool to functional imaging, able to reflect changes in tumour burden and clinical outcomes and with the potential to identify predictive biomarkers.

The main limitations of this work were a single‐institution retrospective design and the small number of patients, and the inclusion of a very heterogeneous patient population, regardless of treatment line. In addition, with growing interest in novel tracers such as prostate‐specific membrane antigen, with higher sensitivity and specificity than other lipid metabolism tracers [33], the use of FCH‐PET/CT is debated. Nevertheless, FCH‐PET/CT is still utilized in clinical practice and has greatly improved the management of prostate cancer patients providing both anatomical information and metabolic information.

## 5 Conclusions

The current prognostic model was built and validated using data routinely collected in mCRPC treated with ARSI in combination with molecular and metabolic data, leading to the identification of patient subsets with very different survival outcomes. This model may be also useful for clinical trial design of new therapeutic approaches in mCRPC. The inclusion of biomarkers with risk assessment models tumour such as hormone‐naive, high‐risk localized cancer. These findings underline the feasibility of combining molecular and functional imaging but require a validation in larger prospective studies leading to future possible standardization and cost‐effectiveness in clinical routine application.

## Conflict of interest

V. Conteduca has served as consultant/advisory board member for Janssen, Astellas, Merck, AstraZeneca and Bayer and has received speaker honoraria or travel support from Astellas, Janssen, Ipsen, Bayer and Sanofi. U. De Giorgi reports personal fees from Astellas, Pharma, Bayer, Novartis and MSD; personal fees and nonfinancial support from Bristol Myers‐Squibb, Ipsen, Janssen, Pfizer and grants and personal fees from Sanofi and Roche and grants from AstraZeneca. No potential conflicts of interest were disclosed by the other authors.

### Peer Review

The peer review history for this article is available at https://publons.com/publon/10.1002/1878‐0261.13120.

### Author contributions

VC conceptualized and designed the study. VC, PC, CL, GG, NB, GP, AF, AA, GS and FM involved in acquisition of data. VC and ES analysed and interpreted the data. VC, GP and UDG wrote, reviewed and/or revised the manuscript. UDG supervised the study.

## Supporting information


**Fig. S1.** Association of plasma tumour DNA (ptDNA) fraction with metastatic sites.
**Fig. S2.** Analysis of plasma tumour DNA (ptDNA fraction) and clinical variable associations.
**Fig. S3.** Linear regression (diagonal line) between maximum standardized total lesion activity (TLA), metabolic tumour volume (MTV), and maximum standardized uptake value (SUVmax).

**Table S1.** Clinical and molecular features of mCRPC patients included in the training and validation cohorts for OS and PFS models.

**Table S2.** Univariate analysis of Overall Survival in the training cohort.
**Table S3.** Univariate analysis of Progression‐Free Survival in the training cohort.

## Data Availability

The data that support the findings of this study are available in the Supporting information of this article and from the corresponding author upon reasonable request.
