# Supplementary material for: Combining liquid biopsy and functional imaging analysis in metastatic castration‐resistant prostate cancer helps predict treatment outcome
Source: Mol Oncol. 2021 Nov 9;16(2):538–48. doi: 10.1002/1878-0261.13120 (PMC8763654; doi:10.1002/1878-0261.13120)
Supplement: Supplementary file 1 — Fig. S1. Association of plasma tumour DNA (ptDNA) fraction with metastatic sites. Fig. S2. Analysis of plasma tumour DNA (ptDNA fraction) and clinical variable associations. Fig. S3. Linear regression (diagonal line) between maximum standardized total lesion activity (TLA), metabolic tumour volume (MTV), and maximum standardized uptake value (SUVmax). [file MOL2-16-538-s002.docx]

**Supplementary Figures for**

**Combining liquid biopsy and functional imaging analysis in metastatic castration-resistant prostate cancer helps predict treatment outcome Running Title:** Plasma tumour DNA and functional imaging in mCRPC

Vincenza Conteduca, Emanuela Scarpi, Paola Caroli, Cristian Lolli, Giorgia Gurioli, Nicole Brighi, Giulia Poti, Alberto Farolfi, Amelia Altavilla, Giuseppe Schepisi, Federica Matteucci, Giovanni Paganelli, Ugo De Giorgi


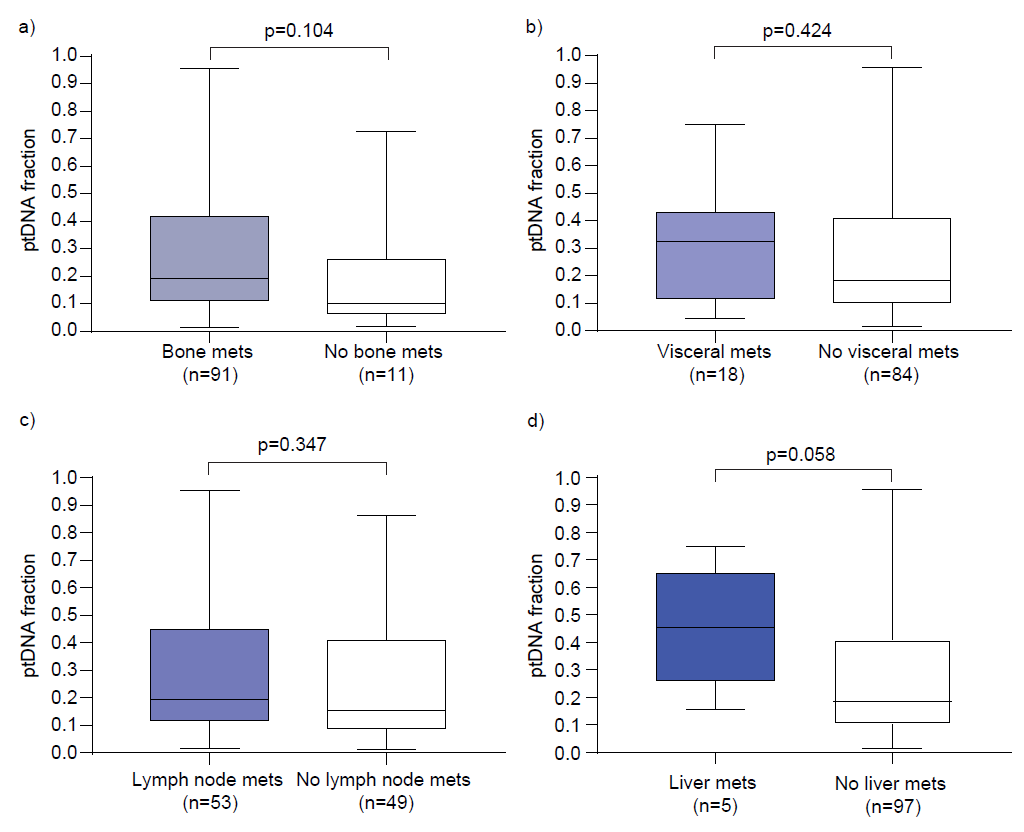


**Supplementary Figure S1. Association of plasma tumour DNA (ptDNA) fraction with metastatic sites.** Correlation of ptDNA level and metastases at (a) bone, (b) visceral, (c) lymph node, and (d) liver. Statistical test used: Wilcoxon-Mann-Whitney test for comparison of plasma tumour DNA (ptDNA) fraction considered as continuous data and metastatic sites. Data from 102 patients were analysed. The left and right sides of the box are the lower and upper quartiles. The box covers the interquartile interval, where 50% of the data is found. The vertical line that split the box in two is the median. The whiskers are the two lines outside the box, that go from the minimum to the lower quartile (the start of the box) and then from the upper quartile (the end of the box) to the maximum.


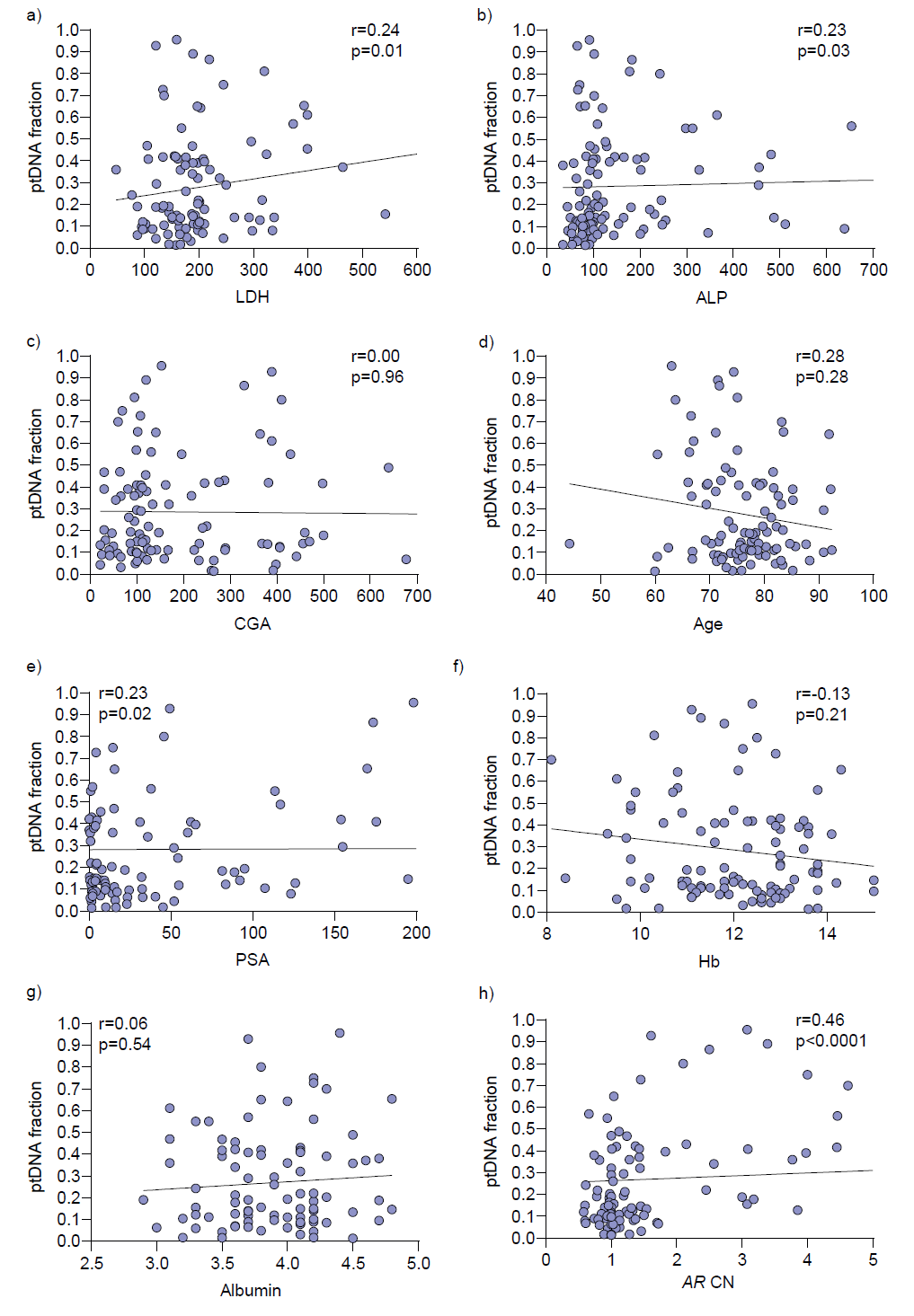


**Supplementary Figure S2. Analysis of plasma tumour DNA (ptDNA fraction) and clinical variable associations.** Correlation of ptDNA level and (a) serum lactate dehydrogenase (LDH), (b) alkaline phosphatise (ALP), (c) serum chromogranin A (CGA), (d) age, (e) prostate specific antigen (PSA), (f) haemoglobin (Hb), (g) albumin, and (h) copy number (CN) of androgen receptor (AR). The relationship between quantitative variables was examined using the linear correlation coefficient (Pearson product moment correlation coefficient). Data from 102 patients were analysed for all variables except for albumin (n=95) and SUVmax (n=101).


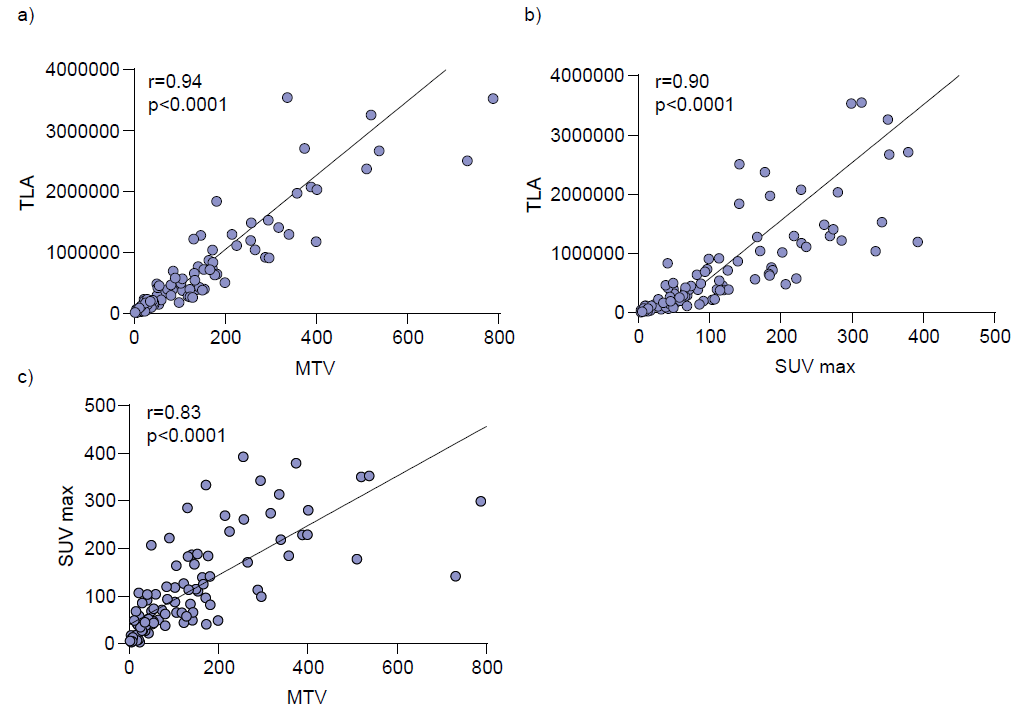


**Supplementary Figure S3. Linear regression (diagonal line) between maximum standardized total lesion activity (TLA), metabolic tumour volume (MTV), and uptake value (SUVmax).** The relationship between quantitative variables was examined using the linear correlation coefficient (Pearson product moment correlation coefficient). Data from 102 patients were analysed for all variables except for albumin (n=95) and SUVmax (n=101).
