# Supplementary material for: Combining liquid biopsy and functional imaging analysis in metastatic castration‐resistant prostate cancer helps predict treatment outcome
Source: Mol Oncol. 2021 Nov 9;16(2):538–48. doi: 10.1002/1878-0261.13120 (PMC8763654; doi:10.1002/1878-0261.13120)
Supplement: Supplementary file 3 — Table S2. Univariate analysis of Overall Survival in the training cohort. Table S3. Univariate analysis of Progression‐Free Survival in the training cohort. [file MOL2-16-538-s001.docx]

**Supplementary Tables for**

**Combining liquid biopsy and functional imaging analysis in metastatic castration-resistant prostate cancer helps predict treatment outcome**

**Running Title:** Plasma tumour DNA and functional imaging in mCRPC

Vincenza Conteduca, Emanuela Scarpi, Paola Caroli, Cristian Lolli, Giorgia Gurioli, Nicole Brighi, Giulia Poti, Alberto Farolfi, Amelia Altavilla, Giuseppe Schepisi, Federica Matteucci, Giovanni Paganelli, Ugo De Giorgi

**Supplementary Table 2. Univariate analysis of Overall Survival in the training cohort**

|  | **N. patients** | **N. events** | **Median OS (months)**  **(95% CI)** | ***P*** | **HR**  **(95% CI)** | ***P*** |
| --- | --- | --- | --- | --- | --- | --- |
| **Overall** | 65 | 63 | 17.6 (11.1-23.1) | - | - | - |
| **Age**, years |  |  |  |  |  |  |
| ≤74* | 37 | 35 | 17.6 (9.2-25.9) |  | 1.00 |  |
| >74 | 28 | 28 | 17.9 (11.1-22.9) | 0.988 | 1.00 (0.60-1.66) | 0.988 |
| **Prostatectomy** |  |  |  |  |  |  |
| No | 38 | 36 | 13.4 (9.9-23.1) |  | 1.00 |  |
| Yes | 27 | 27 | 21.8 (11.21-26.5) | 0.321 | 0.77 (0.46-1.29) | 0.322 |
| **Radical radiotherapy** |  |  |  |  |  |  |
| No | 40 | 39 | 18.6 (11.0-25.3) |  | 1.00 |  |
| Yes | 25 | 24 | 17.6 (8.7-22.5) | 0.517 | 1.18 (0.71-1.98) | 0.518 |
| **Gleason score** |  |  |  |  |  |  |
| 6-7 | 28 | 27 | 22.7 (11.0-26.5) |  | 1.00 |  |
| 8-10 | 30 | 29 | 11.2 (7.4-21.4) | 0.681 | 1.12 (0.65-1.92) | 0.681 |
| **Site of metastasis** |  |  |  |  |  |  |
| No bone | 5 | 5 | 9.8 (2.1-nr) |  | 1.00 |  |
| Bone | 60 | 58 | 17.9 (11.1-23.1) | 0.708 | 1.20 (0.47-3.06) | 0.709 |
| No lymph nodes | 30 | 29 | 18.2 (11.0-27.1) |  | 1.00 |  |
| Lymph nodes | 35 | 34 | 17.6 (9.2-23.7) | 0.642 | 1.13 (0.68-1.86) | 0.643 |
| No visceral | 55 | 53 | 21.8 (11.9-25.3) |  | 1.00 |  |
| Visceral | 10 | 10 | 10.2 (6.3-17.4) | 0.005 | 2.77 (1.33-5.81) | 0.007 |
| **ECOG PS** |  |  |  |  |  |  |
| 0-1 | 63 | 61 | 18.3 (11.0-23.1) |  | 1.00 |  |
| ≥2 | 2 | 2 | 14.4 (11.4-nr) | 0.439 | 1.75 (0.42-7.33) | 0.444 |
| **Presence of pain** |  |  |  |  |  |  |
| No | 59 | 57 | 20.7 (11.1-24.0) |  | 1.00 |  |
| Yes | 6 | 6 | 11.8 (2.1-nr) | 0.013 | 2.92 (1.20-7.11) | 0.018 |
| **Chemotherapy-naive** |  |  |  |  |  |  |
| No | 17 | 15 | 20.7 (7.4-25.3) |  | 1.00 |  |
| Yes | 48 | 48 | 15.6 (11.0-23.7) | 0.615 | 1.16 (0.65-2.09) | 0.615 |
| **Prior therapeutic lines** |  |  |  |  |  |  |
| 1-2 | 43 | 41 | 18.3 (10.6-23.1) |  | 1.00 |  |
| >2 | 22 | 22 | 15.6 (9.4-29.9) | 0.520 | 0.84 (0.49-1.43) | 0.521 |
| **Serum LDH**, U/l |  |  |  |  |  |  |
| <225^#^ | 49 | 47 | 21.4 (13.7-24.0) |  | 1.00 |  |
| ≥225 | 16 | 16 | 9.3 (5.6-17.6) | 0.003 | 2.40 (1.32-4.35) | 0.004 |
| **ALP**, U/l |  |  |  |  |  |  |
| <129^#^ | 51 | 49 | 19.0 (11.4-24.0) |  | 1.00 |  |
| ≥129 | 14 | 14 | 14.5 (2.9-22.5) | 0.062 | 1.76 (0.96-3.23) | 0.066 |
| **NLR** |  |  |  |  |  |  |
| <3^#^ | 34 | 33 | 15.6 (10.6-22.5) |  | 1.00 |  |
| ≥3 | 31 | 30 | 18.3 (9.4-25.9) | 0.453 | 0.82 (0.50-1.36) | 0.454 |
| **Serum CGA**, ng/mL |  |  |  |  |  |  |
| <120^#^ | 27 | 26 | 18.3 (10.6-23.1) |  | 1.00 |  |
| ≥120 | 38 | 37 | 17.5 (9.9-25.3) | 0.405 | 0.80 (0.48-1.35) | 0.407 |

|  | **N. patients** | **N. events** | **Median OS (months)**  **(95% CI)** | ***P*** | **HR**  **(95% CI)** | ***P*** |
| --- | --- | --- | --- | --- | --- | --- |
| **Hemoglobin**, g/dl |  |  |  |  |  |  |
| >12.5^#^ | 25 | 25 | 18.3 (9.9-26.5) |  | 1.00 |  |
| ≤12.5 | 40 | 38 | 16.2 (10.6-23.7) | 0.681 | 0.90 (0.54-1.50) | 0.681 |
| **Serum albumin**, g/dl |  |  |  |  |  |  |
| >4^#^ | 30 | 29 | 18.7 (11.0-25.3) |  | 1.00 |  |
| ≤4 | 30 | 29 | 19.7 (11.0-25.9) | 0.444 | 1.23 (0.72-2.11) | 0.446 |
| **Serum PSA**, ng/dl |  |  |  |  |  |  |
| <32.20* | 32 | 31 | 22.4 (10.6-28.3) |  | 1.00 |  |
| ≥32.20 | 33 | 32 | 11.9 (7.6-21.4) | 0.066 | 1.60 (0.97-2.64) | 0.068 |
| **N. of lesions** |  |  |  |  |  |  |
| <12* | 33 | 31 | 22.9 (11.4-28.3) |  | 1.00 |  |
| ≥12 | 32 | 32 | 14.6 (7.9-20.7) | 0.007 | 2.03 (1.20-3.61) | 0.008 |
| **SUV max** |  |  |  |  |  |  |
| <93.48* | 35 | 33 | 22.9 (11.4-27.7) |  | 1.00 |  |
| ≥93.48 | 29 | 29 | 13.7 (7.6-20.7) | 0.005 | 2.08 (1.23-3.52) | 0.006 |
| **MTV** |  |  |  |  |  |  |
| <102.79* | 35 | 33 | 22.9 (11.9-27.7) |  | 1.00 |  |
| ≥102.79 | 30 | 30 | 12.5 (7.9-18.3) | 0.009 | 1.96 (1.17-3.28) | 0.011 |
| **TLA** |  |  |  |  |  |  |
| <235455* | 28 | 26 | 23.8 (11.0-28.3) |  | 1.00 |  |
| ≥235455 | 37 | 37 | 13.7 (8.6-20.7) | 0.014 | 1.90 (1.13-3.20) | 0.016 |
| **ptDNA** |  |  |  |  |  |  |
| ≤0.201* | 35 | 34 | 24.0 (14.9-28.3) |  | 1.00 |  |
| >0.201 | 30 | 29 | 10.8 (8.6-20.7) | 0.004 | 2.11 (1.25-3.57) | 0.005 |
| ***AR* copy number** |  |  |  |  |  |  |
| Normal | 50 | 48 | 20.2 (11.9-25.3) |  | 1.00 |  |
| Gain | 15 | 15 | 10.6 (3.6-20.7) | 0.008 | 2.23 (1.21-4.10) | 0.010 |

*Median value

^#^Upper normal value

*Abbreviations.* ALP, alkaline phosphatase; *AR*, androgen receptor; CGA, chromogranin A; 95% CI, 95% Confidence Interval; ECOG, Eastern Cooperative Oncology Group; HR, hazard ratio; LDH, lactate dehydrogenase; N, number; NLR, neutrophil-lymphocyte ratio; OS, overall survival; PS, performance status; PSA, prostate-specific antigen.

**Supplementary Table 3. Univariate analysis of Progression-Free Survival in the training cohort**

|  | **N. pts** | **N. events** | **Median PFS (months)**  **(95% CI)** | ***P*** | **HR**  **(95% CI)** | ***P*** |
| --- | --- | --- | --- | --- | --- | --- |
| **Overall** | 65 | 63 | 7.6 (5.6-9.2) | - | - | - |
| **Age**, years |  |  |  |  |  |  |
| ≤74* | 37 | 35 | 7.4 (3.7-9.2) |  | 1.00 |  |
| >74 | 28 | 28 | 8.1 (5.6-10.4) | 0.558 | 0.86 (0.52-1.42) | 0.559 |
| **Prostatectomy** |  |  |  |  |  |  |
| No | 38 | 36 | 7.3 (5.0-8.6) |  | 1.00 |  |
| Yes | 27 | 27 | 9.0 (3.6-15.7) | 0.482 | 0.83 (0.50-1.38) | 0.483 |
| **Radical radiotherapy** |  |  |  |  |  |  |
| No | 40 | 39 | 7.5 (5.2-9.0) |  | 1.00 |  |
| Yes | 25 | 24 | 8.4 (2.7-11.7) | 0.719 | 0.91 (0.54-1.52) | 0.720 |
| **Gleason score** |  |  |  |  |  |  |
| 6-7 | 28 | 27 | 8.6 (5.0-11.0) |  | 1.00 |  |
| 8-10 | 30 | 29 | 5.4 (3.6-9.2) | 0.721 | 1.10 (0.65-1.87) | 0.721 |
| **Site of metastasis** |  |  |  |  |  |  |
| No bone | 5 | 5 | 7.4 (1.5-44.3) |  | 1.00 |  |
| Bone | 60 | 58 | 7.7 (5.0-9.3) | 0.820 | 1.11 (0.44-2.81) | 0.820 |
| No lymph nodes | 30 | 29 | 8.5 (6.5-11.7) |  | 1.00 |  |
| Lymph nodes | 35 | 34 | 7.4 (3.7-9.2) | 0.637 | 1.13 (0.68-1.85) | 0.638 |
| No visceral | 55 | 53 | 7.8 (5.2-9.5) |  | 1.00 |  |
| Visceral | 10 | 10 | 6.7 (2.4-8.4) | 0.302 | 1.43 (0.72-2.84) | 0.305 |
| **ECOG PS** |  |  |  |  |  |  |
| 0-1 | 63 | 61 | 7.5 (5.2-9.2) |  | 1.00 |  |
| ≥2 | 2 | 2 | 9.9 (8.4-11.4) | 0.934 | 0.94 (0.23-3.89) | 0.935 |
| **Presence of pain** |  |  |  |  |  |  |
| No | 59 | 57 | 7.6 (5.2-9.3) |  | 1.00 |  |
| Yes | 6 | 6 | 7.6 (1.5-10.0) | 0.194 | 1.76 (0.74-4.16) | 0.200 |
| **Chemotherapy-naive** |  |  |  |  |  |  |
| No | 17 | 15 | 9.2 (3.6-18.3) |  | 1.00 |  |
| Yes | 48 | 48 | 7.5 (5.0-9.0) | 0.049 | 1.83 (0.99-3.36) | 0.052 |
| **Prior therapeutic lines** |  |  |  |  |  |  |
| 1-2 | 43 | 41 | 7.5 (5.6-9.2) |  | 1.00 |  |
| >2 | 22 | 22 | 8.2 (3.3-11.4) | 0.673 | 1.12 (0.66-1.88) | 0.673 |
| **Serum LDH**, U/l |  |  |  |  |  |  |
| <225^#^ | 49 | 47 | 8.6 (7.2-10.4) |  | 1.00 |  |
| ≥225 | 16 | 16 | 4.5 (2.3-6.0) | 0.0006 | 2.73 (1.50-4.99) | 0.001 |
| **ALP**, U/l |  |  |  |  |  |  |
| <129^#^ | 51 | 49 | 8.4 (6.5-10.0) |  | 1.00 |  |
| ≥129 | 14 | 14 | 5.3 (1.6-9.5) | 0.066 | 1.75 (0.96-3.20) | 0.069 |
| **NLR** |  |  |  |  |  |  |
| <3^#^ | 34 | 33 | 7.4 (4.6-9.3) |  | 1.00 |  |
| ≥3 | 31 | 30 | 8.4 (4.4-11.0) | 0.707 | 0.91 (0.55-1.49) | 0.707 |
| **Serum CGA**, ng/mL |  |  |  |  |  |  |
| <120^#^ | 27 | 25 | 7.5 (4.6-9.3) |  | 1.00 |  |
| ≥120 | 38 | 37 | 7.7 (4.4-11.0) | 0.536 | 0.85 (0.51-1.42) | 0.536 |

|  | **N. pts** | **N. events** | **Median PFS (months)**  **(95% CI)** | ***P*** | **HR**  **(95% CI)** | ***P*** |
| --- | --- | --- | --- | --- | --- | --- |
| **Hemoglobin**, g/dl |  |  |  |  |  |  |
| >12.5^#^ | 25 | 25 | 8.4 (4.4-11.4) |  | 1.00 |  |
| ≤12.5 | 40 | 38 | 7.3 (5.0-9.0) | 0.826 | 0.94 (0.57-1.57) | 0.825 |
| **Serum albumin**, g/dl |  |  |  |  |  |  |
| >4^#^ | 30 | 29 | 8.5 (5.6-13.9) |  | 1.00 |  |
| ≤4 | 30 | 29 | 7.3 (4.4-9.5) | 0.275 | 1.33 (0.79-2.24) | 0.277 |
| **Serum PSA**, ng/dl |  |  |  |  |  |  |
| <32.20* | 32 | 31 | 8.2 (4.4-11.7) |  | 1.00 |  |
| ≥32.20 | 33 | 32 | 7.2 (4.6-9.0) | 0.287 | 1.31 (0.79-2.16) | 0.289 |
| **N. of lesions** |  |  |  |  |  |  |
| <12* | 33 | 31 | 9.2 (7.4-11.7) |  | 1.00 |  |
| ≥12 | 32 | 32 | 5.8 (3.6-8.4) | 0.009 | 1.95 (1.17-3.26) | 0.011 |
| **SUV max** |  |  |  |  |  |  |
| <93.48* | 35 | 33 | 9.2 (5.0-11.7) |  | 1.00 |  |
| ≥93.48 | 29 | 29 | 6.5 (4.4-8.6) | 0.022 | 1.82 (1.08-3.07) | 0.024 |
| **MTV** |  |  |  |  |  |  |
| <102.79* | 35 | 33 | 9.2 (6.8-11.7) |  | 1.00 |  |
| ≥102.79 | 30 | 30 | 6.2 (4.4-8.6) | 0.032 | 1.74 (1.04-2.91) | 0.034 |
| **TLA** |  |  |  |  |  |  |
| <235455* | 28 | 26 | 8.9 (5.0-13.3) |  | 1.00 |  |
| ≥235455 | 37 | 37 | 7.2 (4.6-9.0) | 0.046 | 1.70 (1.01-2.88) | 0.048 |
| **ptDNA** |  |  |  |  |  |  |
| ≤0.201* | 35 | 34 | 9.3 (7.4-11.7) |  | 1.00 |  |
| >0.201 | 30 | 29 | 4.1 (3.0-8.4) | 0.037 | 1.69 (1.02-2.80) | 0.040 |
| ***AR* copy number** |  |  |  |  |  |  |
| Normal | 50 | 48 | 8.6 (6.0-10.4) |  | 1.00 |  |
| Gain | 15 | 15 | 5.6 (1.6-7.5) | 0.009 | 2.22 (1.20-4.09) | 0.011 |

*Median value

^#^Upper normal value

*Abbreviations.* ALP, alkaline phosphatase; *AR*, androgen receptor; CGA, chromogranin A; 95% CI, 95% Confidence Interval; ECOG, Eastern Cooperative Oncology Group; HR, hazard ratio; LDH, lactate dehydrogenase; N, number; NLR, neutrophil-lymphocyte ratio; OS, overall survival; PS, performance status; PSA, prostate-specific antigen.
